# Deep Learning Framework for Controlling Work Sequence in Collaborative Human–Robot Assembly Processes

**DOI:** 10.3390/s23010553

**Published:** 2023-01-03

**Authors:** Pedro P. Garcia, Telmo G. Santos, Miguel A. Machado, Nuno Mendes

**Affiliations:** 1 UNIDEMI, Department of Mechanical and Industrial Engineering, NOVA School of Science and Technology, Universidade NOVA de Lisboa, 2829-516 Caparica, Portugal; 2 Laboratório Associado de Sistemas Inteligentes, LASI, 4800-058 Guimarães, Portugal

**Keywords:** visual assembly task recognition, human–robot collaborative assembly, online class detection, deep learning

## Abstract

The human–robot collaboration (HRC) solutions presented so far have the disadvantage that the interaction between humans and robots is based on the human’s state or on specific gestures purposely performed by the human, thus increasing the time required to perform a task and slowing down the pace of human labor, making such solutions uninteresting. In this study, a different concept of the HRC system is introduced, consisting of an HRC framework for managing assembly processes that are executed simultaneously or individually by humans and robots. This HRC framework based on deep learning models uses only one type of data, RGB camera data, to make predictions about the collaborative workspace and human action, and consequently manage the assembly process. To validate the HRC framework, an industrial HRC demonstrator was built to assemble a mechanical component. Four different HRC frameworks were created based on the convolutional neural network (CNN) model structures: Faster R-CNN ResNet-50 and ResNet-101, YOLOv2 and YOLOv3. The HRC framework with YOLOv3 structure showed the best performance, showing a mean average performance of 72.26% and allowed the HRC industrial demonstrator to successfully complete all assembly tasks within a desired time window. The HRC framework has proven effective for industrial assembly applications.

## 1. Introduction

Quality, efficiency and productivity are three of the most dominant success indicators in the manufacturing industry, and, for this reason, organizations drive a big amount of their investments and dedication to increase and improve these indicators continuously. With this vision, the Industry 4.0 (i4.0) paradigm underlines several approaches with contemporary technologies and ways of thinking, which have been showing that the use of real-time data boosts efficiency, flexibility, productivity, and production quality levels [1,2,3,4] and that leveraging the connectivity of machines within industrial production environments also creates an opportunity for human workers to intuitively collaborate with flexible robotic cyber-physical systems that are required to take care of their own tasks as well as provide assistance to the human when necessary [5], which is the genesis of the broadly called human–robot collaborative (HRC) production or assembly environments that have been one of the topics in the limelight of artificial intelligence (AI) applications in the manufacturing industry [6].

In such HRC environments, the emphasis is usually given to the human worker, and the robot has to adapt its pre-defined assembly tasks or movements on the fly and in accordance with the current progress of the entire assembly process, and/or to the actions of the human, with the goal of maximizing the potential of each one of the partakers, the versatility, and flexibility of the human worker, and the automation and robustness of the robot. For this, it is necessary to develop a communication framework between both sides, i.e., between the human and the robotic system. Thus, the robotic hardware needs to have the capability of recognizing the tasks that are currently being performed and understand abstract concepts, such an assembly sequence, in order to move at the required time instance and to autonomously teamwork with the human worker throughout the entire assembly process.

The coordination and interaction between the human and the robot proposed in the related work of this study can be broadly divided into two main categories according to the source of the data retrieved and processed by the HRC system:

- Human-centered data—action, gesture and sound or voice recognition regarding the human;
- Data regarding the workspace—solely object detection, object tracking, or pose estimation of the components within the workspace or also in coordination with some kind of information regarding the human worker.

The human-centered data category refers to the human worker’s action or activity recognition via processing data as key-points in the body of the human, e.g., human joints [7], visual and inertial sensorial data referring to certain hand gestures or signals [8,9], or even vocal sounds [10,11] that can be used to trigger the robotic part to perform its movements and tasks, and the human related data is usually acquired by means of haptic and inertial sensors [12,13], surface electromyography signals [14,15], brainwave signals, or external visual sensors (e.g., a red–green–blue (RGB) camera or Microsoft’s Kinect sensor) [16]. The task recognition capability is achieved in systems incumbent to this category by correlating the aforementioned types of data with a specific task or set of tasks in a predefined assembly sequence.

Under this scope, human-centered methods were found to be well established for assembly task recognition, as there are several publications and studies on human action/behavior recognition and prediction for enabling a higher level of understanding of the assembly process, i.e., a predefined correlation between certain key movements or actions performed by the human with the entire assembly task sequence. Xiahne et al. [17] proposed a custom three-dimensional (3D) convolutional neural network (CNN), which was a CNN framework built from scratch used to identify a series of images that were dynamic in time. The training dataset comprised seven labeled video segments of each one of the seven different assembly tasks to be carried out performed by human workers and after some testing and tuning, this framework achieved a mean average precision (mAP) of 82% for human action recognition. However, correlating these identified actions with the assembly tasks, recognition was not successful, according to the authors. By processing data collected from a Myo armband equipped with inertial measurement unit (IMU) and surface electromyography (sEMG), Tao et al. [8] proposed a CNN framework also built from scratch and capable of receiving two different inputs, one from the IMU and the other from the sEMG. A custom dataset with six different classes of common assembly activities (e.g., grabbing a tool or tightening a nut using a wrench) was created and a mAP of 89% for action recognition was achieved. To enable action recognition and estimate operation times for repetitive assembly actions, Chen et al. [7] proposed a combined approach of human pose estimation and tool detection. A convolutional posture machine (CPM) model was used to estimate the human joint coordinates and to evaluate whether a tool is being used; two-dimensional (2D) CNN, 3D CNN, and you only look once version 3 (YOLOv3) were compared. To train these networks, a custom dataset was built, and it comprised 39 video clips of 13 operators performing three different types of different assembly actions, which originated 1493 labeled video frames. YOLOv3 performed slightly better on action recognition accuracy than 3D CNN (92.8% against 88.9%) and 2D CNN performed the worst, with a mAP of 82%. An alternative approach to CNNs in order to enable human–robot collaborative assembly was presented by Cheng et al. [18]. They proposed a long short-term memory (LSTM) artificial neural network for human motion classification and target object estimation and trained it over a custom dataset with 50 trials for each action class. The overall classification of the four action classes was only 30%, but the assembly plan recognition accuracy remained higher than 85%. The average time completion time was reduced by 29.1%.

A research area that runs parallel to the HRC but can contribute to its development is cognitive infocommunications (CogInfoCom). Among several research topics that CogInfoCom is dedicated to are the development of complex human–computer interaction (HCI)-based systems and the study of their efficiencies [19]. The most recent studies in this area have revealed that electroencephalography (EEG)-based brain–computer interface (BCI) [20,21], gesture control [22], and eye/eye tracking [23,24] have appeared as the most frequently used alternative communication tools compared to traditional interfaces such as keyboards, mice and touch screens. These alternative communication tools stimulate more of the senses/human brain of the users, providing them with higher rates of attention, performance, and effectiveness [19,20]. Eventually, the same communication tools may bring the same benefits to robotics by integrating new interface systems for HRC.

On the other hand, the HRC assembly environments based on data regarding the workspace category refers to types of systems that only process data related to the current state workspace, i.e., not only directly dependent from the actions of the human worker but also based on the existence or inexistence of already assembled components or other objects (e.g., tools) [10,25], which can be correlated with a certain time instance within the pre-defined assembly sequence and trigger certain commands to the robotic hardware when required. The data in this case is mainly acquired through captured images or video of the workspace by external visual sensors (e.g., RGB or red–green–blue-depth (RGB-D) cameras), and, therefore, it can be considered as a problem of image recognition or object detection. Under this scope, Ze-Hao et al. [26] proposed an integrated assembly assistance system based on faster region-based convolutional neural network (Faster R-CNN) that localized the correct tool to be used in the current task and displays augmented reality (AR) assembly instructions by means of a display device achieving an mAP of 84.7% for tool detection. Despite not having a moving collaborative robotic piece of hardware, this system shared the foundations of a collaborative environment, as the AR instructions would assist sequentially, according to the detected objects within the workspace. With a similar approach, but this time with a collaborative robotic arm, Park et al. [25] proposed an HRC environment with AR as an assistive tool in task assembly and maintenance assistance for and in which the 3D position and pose detection of the real objects were performed by the Faster R-CNN, with ResNet-101 as a backbone network, performing robust object detection and localization with position and angular errors inferior to 0.03 m and 5°, respectively. In [27], the authors proposed a novel integrated mixed reality (as it also used AR instructions) system for safety-aware HRC using deep learning and digital twin generation by processing both visual data from the components and workspace and human skeletal data, calculating the correct minimum safe distance for the human to perform a specific task and providing AR instructions during assembly. A similar approach to this, based on the integration of deep learning models that processed video, skeletal information, and data from wearables that humans were wearing, was proposed by Zhang et al. for context-aware human action recognition and prediction [28]. They expose the effectiveness of the developed method experimentally on a testbed that simulates an assembly environment. High accuracy in both action recognition and prediction was demonstrated.

This study proposes an HRC framework for managing assembly processes that are performed simultaneously or individually by humans and robots. This HRC framework based on deep learning models uses only one type of data, RGB camera data, to make predictions about the collaborative workspace and human action and consequently manage the assembly process.

The remainder of this paper is organized as follows. In Section 2, we present a novel methodology for creating an HRC framework. Section 3 details how each step of the proposed methodology can be implemented in an industrial HRC environment. The results are presented and discussed in Section 4, and the final remarks regarding this study are summarized in Section 5.

## 2. Proposed Approach for Collaborative Human–Robot Assembly

This study proposes an approach for cyber-physical HRC assembly systems using deep learning models as computer vision frameworks. The decisions regarding the exact time instance and which pre-defined movement a robot should perform are integrated in an algorithm developed to manage this system in an automatic manner, i.e., without any direct input from an external party (human or robot), e.g., the human pressing a button to command the robot. Instead, the triggers to command the robot are sent from the HRC framework based on visual information regarding tools and parts, moved in the collaborative workspace and human action.

An HRC system of such topology can be divided into two distinct parts. The first part concerns the development and validation of deep learning models to make predictions about collaborative workspace and human action. On the other hand, the second part concerns the integration of the deep learning model with a management algorithm that synchronously coordinates an assembly process resulting from the teamwork of a collaborative robot and a human worker.

### 2.1. Deep Learning Model

To create a deep learning model capable of making detection/recognition/prediction about the collaborative workspace and human action, the following methodology is proposed:

1. Firstly, the physical HRC workspace where both the human worker and the collaborative robot will co-work is defined. This also comprises defining beforehand the entire assembly sequence regarding the mechanical component to be built, as well as the tools and equipment necessary to conclude the entire assembly process;
2. Subsequently, a large set of images (as large as possible) of objects and human action to be detected inserted into the scenes of interest for the operation of the HRC environment is acquired by a camera, for further training of the deep learning models. It is necessary to account for the importance of not having categorical class imbalance in this set of images, i.e., there should be roughly the same number of instances of each one of the categorical classes to be detected within the entire set of images. The images acquired by the camera are a mixture of frames taken while the human or/and the robot are continuously performing the entire assembly sequence, as well as individual pictures showing a smaller set of components or just an individual categorical class per image;
3. After the acquisition of a large set of images, the manual labelling and definition of bounding boxes for each one of the images with each one of the chosen categorical classes has to be made, concluding in this way the creation of the dataset. The dataset is then divided into training dataset, validation dataset and test dataset. The training and validation datasets are to be used during the training process of a deep learning model and the test dataset to assess and compare its performance;
4. In this step, the structure (backbone) of a deep learning model is created using a CNN architecture. This architecture can be created from scratch, or, alternatively, an existing architecture that has been successful in solving a similar problem can be used and adapted to the desired case;
5. Before training the deep learning model, it is necessary that all the labeled images that make up the datasets are resized to match the image size of the input layer of the deep learning model. In addition, data augmentation is performed on the training and validation datasets in order to quickly create more intra-class variation (e.g., add noise, rotation, translation) and/or artificially increase the number of images in each of the datasets without the need to physically acquire more images;
6. The deep learning model should be trained by a neural network training method having previously selected the training parameters, namely number of epochs, batch size, initial learning ratio, dropout and L2 regularization.

In order to know whether a deep learning model performs well, performance evaluation metrics should be used. For an HRC system such as the one proposed in this study, predicting a positive class is more interesting than predicting a negative class, i.e., it is more relevant to know whether the detected component is actually the correct one than to know whether it is a false or true negative, and also because the problem at hand is the detection of objects from multiple classes rather than a simpler binary classification problem [29], the metrics used to evaluate and compare deep learning models are the accuracy vs recall curve, also known as PR curve, and its mAP. In addition, the detection speed of a deep learning model should be used as an evaluation metric to compare two or more models. This metric can be calculated in several ways: one of them, used in this study, consists in processing with a deep learning model the same image a certain number of times, the average value of the time required to calculate the bounding boxes and labels predicted per image is the result used as the model detection speed.

### 2.2. Management Algorithm for Collaborative Human–Robot Assembly

An algorithm is proposed to manage the assembly process performed simultaneously by the robot and the human. This algorithm receives information from the deep learning model about the collaborative work environment and then decides when and which task the robot should perform. The robot is directly controlled by this decision-making algorithm.

The feasibility of the HRC framework is linked to the class detection performance of the deep learning model and a high level of understanding of the entire assembly process to be performed. The management algorithm correlates the detected classes with the current progress of the assembly process, checking which actions the robot needs to perform and which are already completed, thus allowing visual recognition of the task.

To empower the HRC framework with this feature of a high level of understanding, the following methodology should be applied:

1. Based on an assembly sequence, specific tasks are assigned to the human and the robot. Emphasis is placed on the human worker, so the robot performs assistive tasks, such as picking up tools or part assemblies out of the human’s reach and bringing them to the worker or mounting them on other parts;
2. The tasks allocated to the robot must be taught to it. The robot must execute these tasks in an uncoupled manner, which means that the robot can execute any task at any time, without imposing on the robot that the execution of a task is dependent on the previous execution of another task. The sequence will be imposed by the decision-making algorithm and not by the robot itself. Each task must be taught to the robot in a way that ensures the safety of the human, i.e., the robot must change position with reduced accelerations and final speeds, with a trajectory that avoids collisions and maintains a minimum safety distance from the human worker;
3. The next step is to define which parts/sets of parts, tools and human actions, detected by the deep learning model, as well as which conditions of the robotic cell (e.g., joint positions or robot speeds) should act as triggers to command the robot to perform a task. In order to ensure high reliability to the HRC framework, classes recognized by the deep learning model must be recognized in at least *m* frames when *n* frames are processed, where *m* < *n*. Define the *m* frames;
4. Based on the previous points, the management algorithm is implemented using a programming language. A generic structure for this algorithm is suggested in Algorithm 1.

**Algorithm 1:** Management Algorithm  1: Initialize communication between management algorithm and robot controller  2: *camera* ← Set up the camera  3: *model* ← Load deep learning model  4: *storage_detected_classes* ← Reset the classes detected in the last *n* frames  5: *tasks_performed* ← Reset the registered tasks as performed by the robot  6: *num_frames* ← 0 Reset the frame count captured by the camera  7: *running_flag* ← **true**  8: **while** (*running_flag*)  9:  *image* ← Capture_Image (*camera*)10:  *detected_classes* ← *model* (*image*)11:  *storage_detected_classes* ← Update (*detected_classes*)12:  *tasks_performed* ← The robot provides feedback about task execution13:  *num_frames* ← *num_frames* + 114:  **if** (*num_frames* == *n*)15:   **if** (Existence **and** non-existence of certain key categorical classes within the workspace detected in *m* frames (*storage_detected_classes*) **and** certain tasks performed by the robot were **and** were not performed (*tasks_performed*) as required by robot task 1)16:     Command the robot to perform the robot task 117:     *num_frames* ← 018:     *storage_detected_classes* ← Reset19:    **elseif** (Existence **and** non-existence of certain key categorical classes within the workspace detected in *m* frames (*storage_detected_classes*) **and** certain tasks performed by the robot were **and** were not performed (*tasks_performed*) as required by robot task 2)20:     Command the robot to perform the robot task 221:     *num_frames* ← 022:     *storage_detected_classes* ← Reset23:    **elseif** (…)24:     …25:    **elseif** (All *k* robot tasks were performed (*tasks_performed*))26:     *tasks_performed* ← Reset27:    **endif**28:    **if** (*num_frames* == *n*)29:     *num_frames* ← *num_frames*-130:    **endif**31:   **endif**32:   *running_flag* ← Check if the human has turned off the HRC application 33: **endwhile**

The initial steps of the HRC framework are initializing all the required software for the HRC interface, setting up the camera to continuously acquire images and resizing its output to be used as an input by the deep learning model (*model*). Then, the deep learning model processes and performs class detection on these input frames and the detection results (labels and bounding boxes) of a *n* number of frames are stored in a temporary variable (*storage_detected_classes*). Afterwards, it is necessary to evaluate whether in a certain *m* number of these *n* frames (where *m* < *n*) the predefined classes and other conditions of the robotic cell (*tasks_performed*) are satisfied (e.g., final joint positions of a robotic arm)—where these conditions and detected classes are linked to a specific robot task from the predefined assembly sequence. If all the conditions of the robotic cell (*tasks_performed*) and the minimum number of detected classes instances (*storage_detected_classes*) for a specific task are satisfied, the HRC framework commands the collaborative robot to perform a robot task and makes the sequence move forward to the next task, overriding the temporary variable *storage_detected_classes* with the detections of the *n* frames with new ones and proceeding to new detections. On the other hand, if all the conditions of the robotic cell and the minimum number of detected classes instances are not satisfied, no commands are triggered in the robot and the temporary variable *storage_detected_classes* with the detections of the *n* frames is overridden with new detections continuously until all the conditions of the robotic cell for a certain robot task are satisfied. Once the last task in the assembly sequence is complete, the HRC framework resets all its variables to start a new assembly sequence. At any time, the human worker can stop the assembly sequence by pressing a physical or virtual button. Thus, the HRC framework receives this information (*running_flag*) and stops the assembly sequence.

## 3. Human–Robot Collaborative Assembly Environment

**Figure 1:**
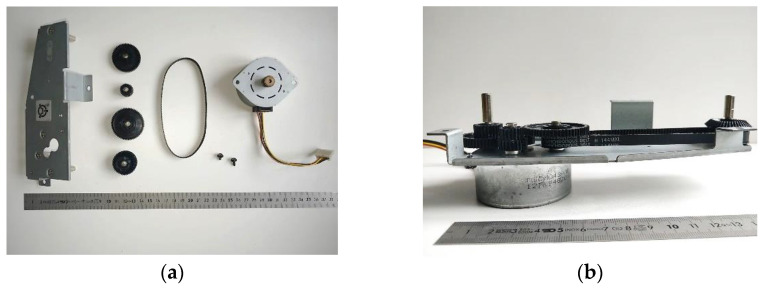
Mechanical component: (**a**) disassembled in parts; (**b**) assembled.

**Figure 2:**
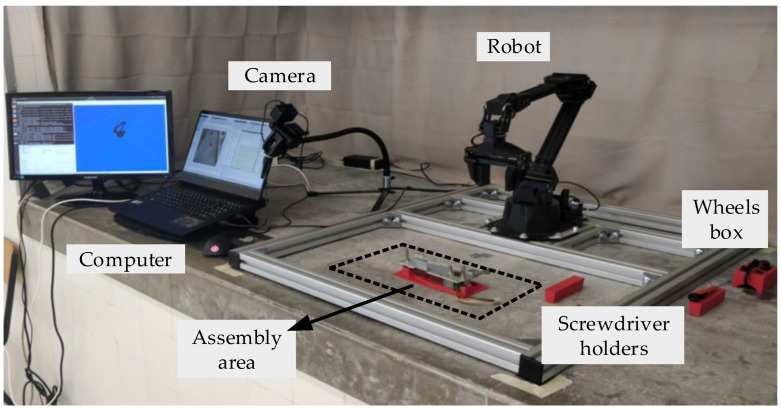
HRC environment.

**Figure 3:**
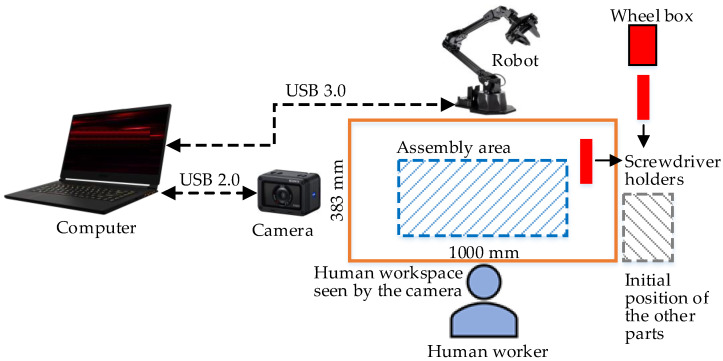
Architecture of the HRC robotic cell that constitutes the HRC environment.

To test the ability of the HRC framework, suggested in Section 2, to operate in HRC environments, the HRC framework was implemented to manage the assembly process of the mechanical component illustrated in Figure 1. This HRC environment consists of a ViperX 300 robot, a Sony DSC-RX0M2G camera, and a laptop equipped with i7-10870H CPU, NVIDIA GeForce RTX 3070 8 GB GDDR6 GPU and 16 GB DDR4 RAM. The layout of the HRC environment is illustrated in Figure 2. The architecture of the HRC robotic cell that constitutes the HRC environment is depicted in Figure 3. All software was developed in the MatLab programming language.

### 3.1. Deep Learning Model Creation

**Figure 4:**
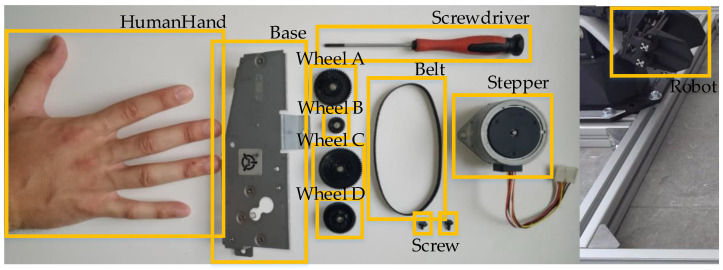
Categorical classes (Base, Stepper, Belt, Robot, Screwdriver, Screw, HumanHand, Wheel_A, Wheel_B, Wheel_C and Wheel_D).

After creating the HRC environment, an image dataset was created with the camera observing the workspace. This dataset consisted of 3858 images with an initial size of 576 × 1024 pixels each. After this step, all acquired images were manually labeled to create a training dataset consisting of 11 different categorical classes. To avoid class imbalance, images containing about 1100 instances of each categorical class were acquired. These 11 categories were labeled as: Base, Stepper, Belt, Robot, Screwdriver, Screw, HumanHand, Wheel_A, Wheel_B, Wheel_C and Wheel_D. The 11 categorical classes are displayed in Figure 4.

Four deep learning models were built to perform the recognition of the above-mentioned categorical classes. These four models were built from existing deep learning models, which belong to the Faster R-CNN [30] and the YOLO [31] branches and were successful in recognizing parts and objects similar to the categorical classes proposed in this study. The backbone of the deep learning models built are Faster R-CNNs ResNet-50 and ResNet-101 [32], YOLOv2 [33] and YOLOv3 [34], i.e., two models based on the Faster R-CNN architecture and two models based on the YOLO branch. The main characteristics of the four deep learning models are presented in Table 1. All deep learning models receive an image as input data. In the first step, a model proposes potential regions of the image where eventually known classes by the model may be present. In the second step, the potential image regions are classified by the model to find out which classes are present in the regions. Finally, the model outputs the regions that have classes known to the model and which class is present in each region. The four different deep learning models were subjected to a transfer learning process, i.e., the existing models were trained with the dataset created for this HRC assembly application, thereby adjusting its weights and biases for detection of the desired classes.

Before the deep learning models were trained, the initial dataset with the 3858 images was randomly divided into training (80% of the initial dataset), validation (10%) and test (10%) datasets and kept the same for the four models. As with the creation of the initial dataset, the image splitting that created these three datasets was performed with consideration of the need for class balance, i.e., approximately the same number of instances of each the different categorical classes. The training and validation datasets were given use during the process of training and the test dataset was used to assess the performance of each of the models. In addition, data augmentation was performed on the training dataset, where 50% of all the images were randomly flipped horizontally in an effort to address issues such as overfitting and the immediate increase in intra-class variation within the training dataset.

The four deep learning models were trained using the training parameters shown in Table 2. The number of epochs and the mini-batch size were decided on a trial-and-error process to not overshoot the GPU memory available during training.

### 3.2. Implementation of the Management Algorithm

The management algorithm, presented in Section 2.2, was applied to the collaborative environment under test to perform the assembly of the mechanical component represented in Figure 1. This algorithm has the function of enabling the robot to autonomously decide when to perform a certain task to assist the human in performing his/her tasks.

To implement the algorithm, first the sequence of assembly tasks and the topology was defined, as well as the additional equipment needed in addition to the parts to be assembled, namely an assembly support, a screwdriver and a box to store the cogwheels and the belt. The sequence of assembly tasks is shown in Table 3.

Once the assembly sequence was established, the tasks that the robot was expected to do were defined. The robot was programmed to perform the four different robot tasks described in Table 4, with the goal of assisting the human during the assembly process, e.g., to pick up a tool that was out of the human’s reach.

The aforementioned robot tasks were only to be performed in accordance with the progress of the assembly tasks performed by the human. It was defined that the robot would:

- Perform task R1 only when the human was performing the insertion of the screws in place (task H3);
- Perform task R2 only when the human started screwing (task H4);
- Perform task R3 only when the human turned the half-assembled mechanical component upwards (task H5);
- Perform task R4 only when all the components were assembled (completed task H6).

To correlate the video data captured by the camera with the desired higher level of understanding of the assembly progress, the algorithm presented in Section 2.2, Algorithm 1, was implemented to this HRC robotic cell. This algorithm allowed the deep learning model to process each frame captured by the camera in an online loop, and whenever certain key parts would be correctly detected, the robot would know when to perform one of the four pre-defined robot tasks. The implementation of the algorithm is schematically represented in Algorithm 2. In the implementation of this algorithm, the number of frames, *n*—from which the detection results would be stored in a temporary variable—was 10, and regarding the robot tasks R1, R2, R3, and R4 (*k* = 4), the values of *m* were 7, 5, 3 and 2, respectively.
**Algorithm 2:** Implementation of the Management Algorithm to Control the HRC Robotic Cell  1: Initialize communication between management algorithm and robot controller  2: *camera* ← Set up the camera  3: *model* ← Load deep learning model  4: *storage_detected_classes* ← Reset the classes detected in the last *n* frames  5: *tasks_performed* ← Reset the registered tasks as performed by the robot  6: *num_frames* ← 0 Reset the frame count captured by the camera  7: *running_flag* ← **true**  8: **while** (*running_flag*)  9:  *image* ← Capture_Image (*camera*)10:  *detected_classes* ← *model* (*image*)11:  *storage_detected_classes* ← Update (*detected_classes*)12:  *tasks_performed* ← The robot provides feedback about task execution13:  *num_frames* ← *num_frames* + 114:  *n* ← 1015:  **if** (*num_frames* == *n*)16:   **if** (Base **and** Stepper have been detected in at least 7 of the 10 frames (*storage_detected_classes*) **and** R1 has not yet been performed (*tasks_performed*))17:     Command the robot to perform the robot task 118:     *num_frames* ← 019:     *storage_detected_classes* ← Reset20:    **elseif** (Base **and** Screw have been detected in at least 5 of the 10 frames (*storage_detected_classes*) **and** R2 has not yet been performed (*tasks_performed*) **and** R1 has been performed (*tasks_performed*))21:     Command the robot to perform the robot task 222:     *num_frames* ← 023:     *storage_detected_classes* ← Reset24:    **elseif** (The Base has been detected **and** neither the Bolt **nor** the Stepper has been detected in at least 3 of the 10 frames (*storage_detected_classes*) **and** R3 has not yet been performed (*tasks_performed*) **and** R1 **and** R2 have been performed (*tasks_performed*))25:     Command the robot to perform the robot task 326:     *num_frames* ← 027:     *storage_detected_classes* ← Reset28:    **elseif** (The Base **and** the Belt **and** the Wheels A, B, C **and** D have been detected in at least 2 of the 10 frames (*storage_detected_classes*) **and** R4 has not yet been performed (*tasks_performed*) **and** R1 **and** R2 **and** R3 have been performed (*tasks_performed*))29:     Command the robot to perform the robot task 430:     *num_frames* ← 031:     *storage_detected_classes* ← Reset32:    **elseif** (R1 **and** R2 **and** R3 **and** R4 have already been performed (*tasks_performed*))33:     *tasks_performed* ← Reset34:    **endif**35:    **if** (*num_frames* == *n*)36:     *num_frames* ← *num_frames*-137:    **endif**38:   **endif**39:   *running_flag* ← Check if the human has turned off the HRC application 40: **endwhile**

## 4. Results and Discussion

Two types of testing were performed to evaluate the performance of the HRC framework. In one type of test, the performance of the deep learning model to process isolated frames was evaluated. In the other type of test, the performance of the entire HRC framework was evaluated. For this, 50 units of the mechanical component illustrated in Figure 1 were assembled at the HRC environment.

### 4.1. Deep Learning Model Performance

**Figure 5:**
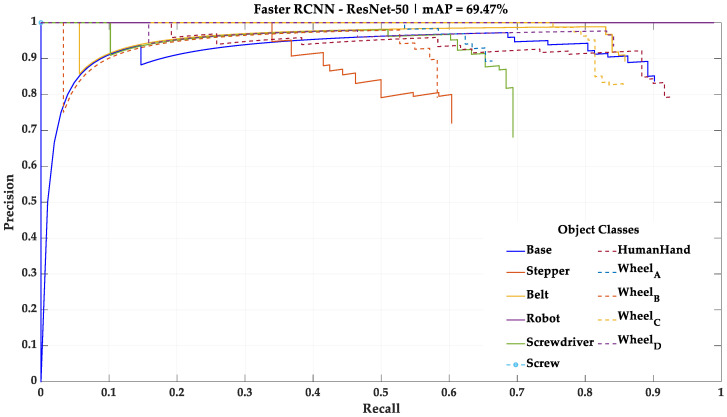
Result of the test performed with the Faster R-CNN—ResNet-50 model using the mini-batch size training parameter of 32.

**Figure 6:**
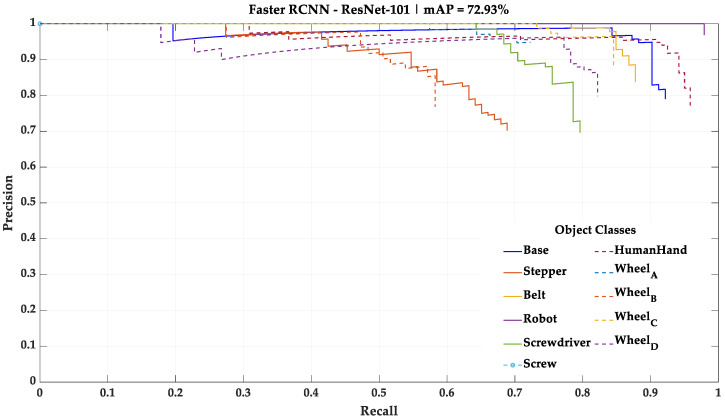
Result of the test performed with the Faster R-CNN—ResNet-101 model using the mini-batch size training parameter of 32.

**Figure 7:**
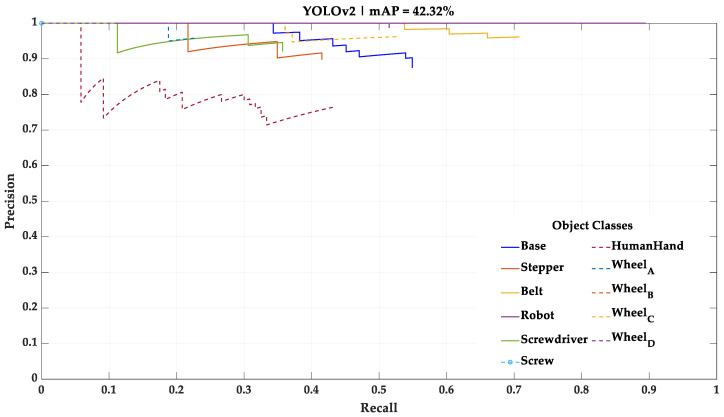
Result of the test performed with the YOLOv2—Darknet-19 model using the mini-batch size training parameter of 32.

**Figure 8:**
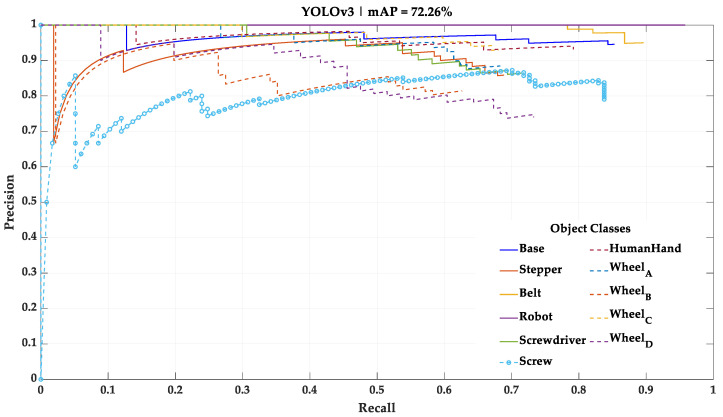
Result of the test performed with the YOLOv3—Darknet-53 model using the mini-batch size training parameter of 32.

As stated in Section 3.1, 10% of the initial dataset was not used during the training of the deep learning models. However, it was used as a test dataset for performance assessment and comparison between the four different deep learning models. In this test, each deep learning model processes the test dataset, one frame at a time, and detects the categorical classes present. The results of this test using the mini-batch size training parameter of 32, the one that yielded the best results, are shown in Figure 5, Figure 6, Figure 7 and Figure 8 in the form of PR curves and mAP. These PR curves express the trade-off between precision and recall for different threshold values and for each of the deep learning models. A good classifier is one that showcases a high area under the curve referring to each of the categorical classes, as well as an approximately horizontal trend throughout the different precision and recall values. The area of the curve for a single categorical class in a perfect classifier is equal to one.

For this study, the mAP with an intersection over union (IoU) of 0.5—a common mAP metric—was considered for the further performance comparison. A condensed overview of the mAP of each one of the deep learning models with this IoU is presented in Table 5.

According to these results, the mini-batch size training parameter that led to the best results in all deep learning models was 32. Thus, this study continued using the four deep learning models trained with the mini-batch size training parameter of 32. The two best performing deep learning models, taking mAP as a metric, were the Faster R-CNN architecture with ResNet-101 as backbone network and YOLOv3 architecture with Darknet-53 as backbone network. Both models exposed an approximately constant precision level for different levels of recall (horizontal trendline), meaning that both are robust for the test data. However, while the ResNet-101 model showed a better understanding regarding the differences between Wheels_A, B, C and D, the Darknet-53 model showed a fair understanding of these differences and was the only model able to detect the small Screw categorical class, which was necessary to be detected for the correct implementation of the HRC framework.

A test was conducted to measure the detection speed of each deep learning model that had been trained with the mini-batch size training parameter of 32. This test consists in measuring the time required for a deep learning model to detect 50 times the categorical classes contained in the same image. At each measurement, the time required to detect the labels, bounding boxes and calculate the confidence of each label was counted. The average detection time per image metric was calculated by dividing the previous recorded time by 50. The results of this test can be seen in Table 6.

When analyzing these results, it is necessary to remember that the detection speed is not only related to the model’s architecture but also deeply related to the input image size of the detector, i.e., higher input sizes require higher computational resources, which explains why the YOLOv3 detector has the slowest detection speed compared to the others, while processing image sizes two to three times higher. However, comparing YOLOv2—Darknet-19 to Faster R-CNN—ResNet-50 and Faster R-CNN—ResNet-101,which have roughly similar image input sizes, the advantage of using YOLOv2-based architectures over Faster R-CNN architectures is evident for online applications, such as similar HRC systems, where a higher order of magnitude of detection speed contributes to better system performance.

### 4.2. Human–Robot Collaborative Framework Performance

**Figure 9:**
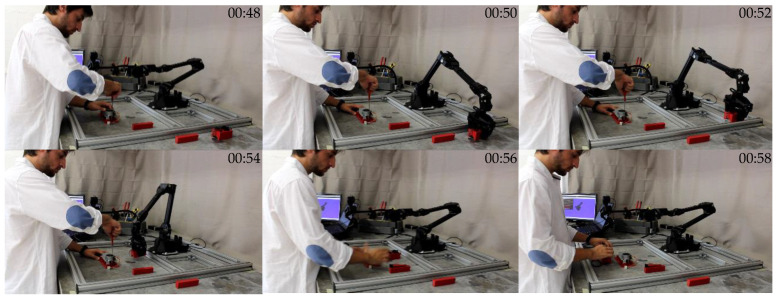
Video frames of the robot being automatically triggered to pick the wheel box and place it on the HWS (task R2) while the screwing task performed by the human is in progress (task H4). The video can be seen in the Supplementary Materials.

Each deep learning model, trained with the mini-batch size training parameter of 32, was integrated into the management algorithm as described in Section 3.2, thus forming four different HRC frameworks and tested in the collaborative environment described in Section 3. The assembly of the mechanical component shown in Figure 1 was repeated 50 times using each of the HRC frameworks. A sequence of images illustrating the moment where the HRC framework automatically triggered the robot to pick the wheel box for the human, while the human worker was performing the screwing task simultaneously, is shown in Figure 9.

The results of this experiment demonstrated that the HRC framework equipped with the YOLOv3 deep learning model allowed performance of the entire assembly sequence of the mechanical component. All the classes needed to control the work sequence in the assembly of the mechanical component were successfully recognized by the YOLOv3 model. In addition, this first HRC framework allowed the human to execute his/her tasks continuously, i.e., the human assembled the nine parts that make up the mechanical component, illustrated in Figure 4, without any situation occurring where the human would stand still waiting for the robot to finish a task so that the human could execute another task afterwards. Unlike this first HRC framework, all the others, equipped with the other deep learning models, only allowed the robot to perform R1 task by failing to detect the conditions for the R2 task to begin. This is because these three HRC frameworks were equipped with deep learning models that never detected the Screw category class, which was necessary for the assembly process management sequence to proceed. The four HRC frameworks allowed all tasks performed by the robot to be performed in the desired order and at the time expected by the human and no unwanted occurrences were recorded.

Based on this experiment, it can be stated that:

- The YOLOv3 deep learning model allowed constitution of a successful HRC framework for managing the sequence of tasks performed by a robot to assemble a mechanical component;
- The HRC framework was capable of triggering the robot to perform its tasks at the exact moment needed, sequentially assisting the human throughout the assembly process;
- The HRC framework made it possible for a human co-working together with a robot within the same workspace;
- The HRC framework proof-of-concept worked effectively and successfully in assembling mechanical parts.

## 5. Conclusions

In this study, four different HRC frameworks were proposed to command a robot to assist a human in assembly processes. A management algorithm and four different deep learning models that have the Faster R-CNN with ResNet-50, the Faster R-CNN with ResNet-101, the YOLOv2 with Darknet-19, and the YOLOv3 with Darknet-53 models as their base structure were used to build the four HRC frameworks. These deep learning models detect the categorical classes present in the HWS and provide these detections to the management algorithm that allows for a higher-level of understanding of the entire assembly sequence and commands the robot accordingly.

The YOLOv3-based deep learning model was best suited for visual task recognition due to the following reasons:

- The best performing deep learning models in terms of mAP values were the models based on YOLOv3 and Faster R-CNN with ResNet-101 (72.26% and 72.93%, respectively). However, YOLOv3 was the only model capable of detecting the Screw categorical class, which was important for the proper functioning of the HRC framework;
- Although the YOLOv3-based model had a similar mAP to that presented by the Faster R-CNN with ResNet-101 model and showed in comparison a worse ability in distinguishing the Wheel_A, Wheel_B, Wheel_C and Wheel_D categorical classes, the YOLOv3-based model was the only model able to detect the Screw class. Due to the nature of the assembly sequence defined for the selected mechanical component, it was determined as more important to correctly detect the Screw categorical class, rather than to perfectly distinguish all categorical classes pertaining to the cogwheels;
- The YOLOv3-based model has the advantage of requiring fewer computational resources and having a faster detection speed, due to the smaller number of layers and parameters comprising the model, than the other deep learning models discussed in this study.

The management algorithm granted the HRC framework a higher level of understanding of the entire assembly sequence, as it correlated the categorical classes detected in a given time instance with the progression of the assembly tasks, commanding the robot as needed and correctly. The HRC framework equipped with the YOLOv3-based deep learning model was capable of managing the entire HRC assembly process, where the robot was automatically commanded to move in the required time instance according to the current state of the assembly process.

Thus, this study outputted an efficient and feasible management methodology for HRC assembly processes. An HRC framework in which task detection is performed solely by taking visual data from an RGB camera as input, which is subsequently processed and analyzed by a YOLOv3-based deep learning model and, according to the current state of the assembly process, i.e., for which parts are already correctly assembled, commands the robot to perform its tasks on the fly.

## Figures and Tables

**Table 1:** Main characteristics of deep learning models.

Architecture	Faster R-CNN	Faster R-CNN	YOLOv2	YOLOv3
Backbone	ResNet-50	ResNet-101	Darknet-19	Darknet-53
Input layer size in pixels [height width]	[224 × 396]	[224 × 396]	[256 × 256]	[608 × 608]
Pretraining dataset	ImageNet [35]	ImageNet [35]	ImageNet [35]	COCO [36]

**Table 2:** Parameters used to train the four deep learning models.

Training Parameters	Value
Solver	Sgdm
Number of epochs	50
Mini-batch size	16/32/64
Initial learning rate	1 × 10^−4^
Dropout	Yes
L2 regularization	1 × 10^−4^
Validation dataset	Yes

**Table 3:** Assembly task sequence.

Task Number	Task Description	Requirements
H1	Mount the bottom of the Base part on the assembly support.	Assembly support
H2	Mount the Stepper on the Base.	N/A
H3	Insert the two Screws in the correct holes to fix the Stepper to the Base.	N/A
H4	Screw the Screws with the Screwdriver.	Screwdriver
H5	Turn the semi-assembled mechanical component upside down (top).	N/A
H6	Assemble the Wheels A, B, C and D and the Belt.	Cogwheel and belt storage box

**Table 4:** Tasks performed by the robot.

Robot Task	Robot Task Description
R1	Pick up the screwdriver from outside the human workspace (HWS) and place it in the screwdriver holder in the HWS.
R2	Pick up the wheel box from outside the HWS and place it in a specific location inside the HWS.
R3	Pick up the screwdriver placed in the inner screwdriver holder, inside the HWS, and carry it to the outer holder, outside the HWS.
R4	Pick up and take away the empty wheel box to the initial position outside the HWS.

**Table 5:** Mean average precision of each deep learning model with a 0.5 IoU.

Model	Mini-Batch Size	mAP_50_ (%)
	16	62.76
Faster R-CNN—ResNet-50	32	69.47
	64	66.34
	16	69.53
Faster R-CNN—ResNet-101	32	72.93
	64	71.43
	16	38.82
YOLOv2—Darknet-19	32	42.32
	64	40.61
	16	67.34
YOLOv3—Darknet-53	32	72.26
	64	70.75

**Table 6:** Detection speed of each deep learning model trained with the mini-batch size training parameter of 32 (GPU processing). Tests performed with 50 trials on a single image.

Deep Learning Model/Input Image Size	Average Detection Time per Image [s]	Standard Deviation [s]
Faster R-CNN—ResNet-50 [224 × 396]	0.346	0.043
Faster R-CNN—ResNet-101 [224 × 396]	0.403	0.036
YOLOv2—Darknet-19 [256 × 256]	0.031	0.009
YOLOv3—Darknet-53 [608 × 608]	0.551	0.060

## Data Availability

The data presented in this study are available on request from the corresponding author.

## Supplementary Materials

The following supporting information can be downloaded at: https://www.mdpi.com/article/10.3390/s23010553/s1, Video S1: Collaborative human-robot assembly process.
